# Application and Prospect of Strapdown Inertial Navigation System in Coal Mining Equipment

**DOI:** 10.3390/s24216836

**Published:** 2024-10-24

**Authors:** Minfu Liang, Daqian Zheng, Kewei Li, Xinqiu Fang, Gang Wu

**Affiliations:** 1 School of Mines, China University of Mining and Technology, Xuzhou 221116, China; liangmf2014@cumt.edu.cn (M.L.); ts23020134p31@cumt.edu.cn (K.L.); fangxinqiu@cumt.edu.cn (X.F.); wu007gang007@cumt.edu.cn (G.W.); 2 Research Center of Intelligent Mining, China University of Mining and Technology, Xuzhou 221116, China

**Keywords:** inertial navigation, coal cutter, integrated navigation

## Abstract

In recent years, with the rapid construction of a safe, clean, efficient and sustainable modern energy system, the intelligence of coal mining has become the inevitable direction of the development of the coal mining industry. The intelligence of coal mining system and equipment is the core of intelligent mining, and the positioning technology of mining equipment is the key to underground intelligent mining. The strapdown inertial navigation system can make up for the shortcomings of traditional GPS positioning systems and laser positioning because of its strong anti-interference, high precision and real-time monitoring, and ability to carry out real-time dynamic positioning of mining equipment in underground coal mines. This paper briefly introduces the basic principle of the strapdown inertial navigation system, analyzes the application of the existing strapdown inertial navigation system in mining equipment, and lists and analyzes the research status of the improvement strategy of the inertial navigation system, such as zero speed correction and integrated navigation technology. Finally, by analyzing the application and future development trend of inertial navigation systems in mining equipment, this paper provides more data and method support for the positioning of mining equipment in the future.

## 1. Introduction

**Figure 1:**
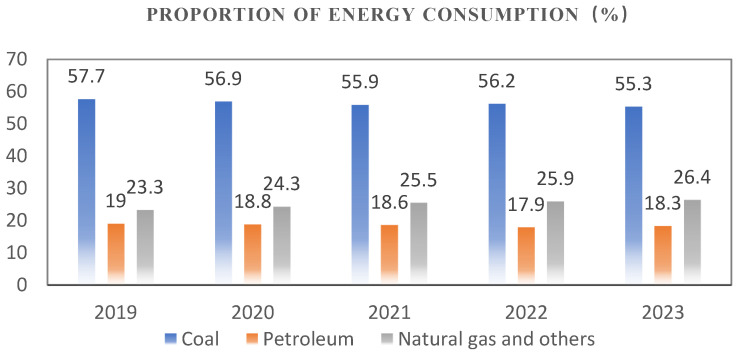
Energy consumption structure chart in 2019–2023.

**Figure 2:**
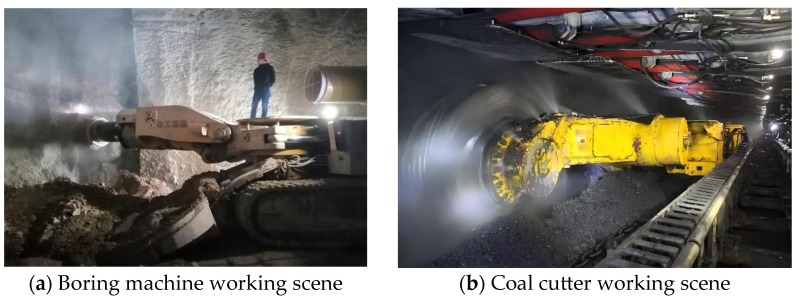
Excavation equipment work site.

In recent years, with the rapid development of a secure, clean, efficient, and sustainable modern energy system by the country, which is shown in Figure 1, the coal demand has declined. In 2023, China’s coal production reached 4.71 billion tons, an increase of 3.4% year-on-year [1]. In order to achieve substantial and effective development of the coal industry, the intelligentization of coal mining has become a necessity and an inevitable direction for the development of the coal industry [2]. Within the intelligentization standard system [3] established by the Wang Guofa Court scholar, intelligent systems and equipment are critical technologies for coal mine intelligence. In underground mining, intelligent systems and equipment include tunneling systems, extraction systems, transport systems, and ventilation systems. Among these, the tunneling and extraction systems, which pertain to the intelligentization of mining equipment, are particularly significant. Accurate positioning and navigation of underground equipment is a prerequisite for the intelligentization of mining tools. However, the operation of these tools often generates significant amounts of dust and water vapor, complicating the working environment. Additionally, as shown in Figure 2, the operation of coal cutters and tunneling machines produces strong vibration signals, which can severely interfere with precise positioning. Therefore, achieving accurate positioning for mining equipment poses significant challenges.

With the continuous development of mining technology, the increasing depth of coal mines, and the complexity of mining environments, the positioning and attitude control technology of excavation equipment is playing an increasingly important role in mining production. This technology refers to the precise control of the posture and position of excavation equipment through various sensors and control systems, ensuring safe and stable operations. In light of the challenges and issues faced by excavation equipment, many scholars, both domestically and internationally, have conducted extensive research and practices. Various attempts have been made regarding existing positioning and attitude control technologies, as reflected in the comparative analysis shown in Table 1.

In comparison to other attitude and positioning technologies, inertial navigation systems can independently complete positioning and attitude calculations without the need for external signal sources, thus offering higher accuracy and stability [4]. Meanwhile, global navigation satellite systems and optical navigation technologies often face challenges in the complex environments of underground coal mines. Mining equipment is frequently disturbed by geological conditions, low visibility, and confined spaces, leading to difficulties in achieving precise positioning and attitude determination. Inertial navigation systems can effectively suppress interference signals through built-in filtering algorithms, enhancing the system’s robustness and anti-interference capabilities, which ensures that equipment can reliably carry out positioning and attitude control tasks.

Due to the numerous advantages of inertial navigation systems in underground operations, the autonomy and intelligence of mining equipment have been enhanced, reducing reliance on manual operations, which has led to the widespread application of these systems in increasingly automated and intelligent mining tools. This article provides a systematic overview of the use of strapdown inertial navigation technology in coal mining equipment, elaborating on its technical principles, applications, and improvement methods. It discusses the progress of strapdown inertial navigation systems in coal mines and analyzes the key issues and solutions related to pose estimation and positioning of mining equipment.

## 2. Basic Principle of Strapdown Inertial Navigation System

**Figure 3:**
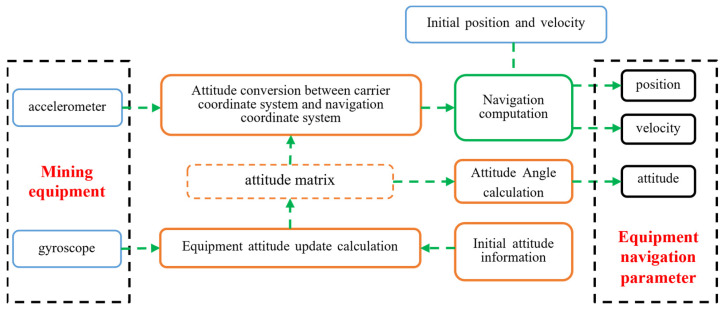
Operational attitude sensing technology of mining equipment based on strapdown inertial navigation system.

The strapdown inertial navigation system directly connects inertial sensors (including accelerometers and gyroscopes) to the moving platform. The acceleration and angular velocity of the platform are measured by the accelerometers and gyroscopes, respectively. By integrating these data with the platform’s initial position, velocity, acceleration, and angular velocity, various navigation parameters such as position, speed, and attitude can be calculated [5,6,7], as illustrated in Figure 3. The strapdown inertial navigation system offers several advantages, including compact size, lightweight, high accuracy, reliability, real-time performance, and independence from external information [8]. The core component, the gyroscope, is typically chosen for its short response time, wide dynamic range, high reliability, strong environmental adaptability, and ease of maintenance. Optical gyroscopes, such as laser gyroscopes and fiber optic gyroscopes, are commonly used. The emergence of these high-precision inertial sensors and the ongoing optimization of attitude calculation algorithms enable strapdown inertial navigation systems to achieve more accurate and reliable positioning in complex environments.

**Figure 4:**
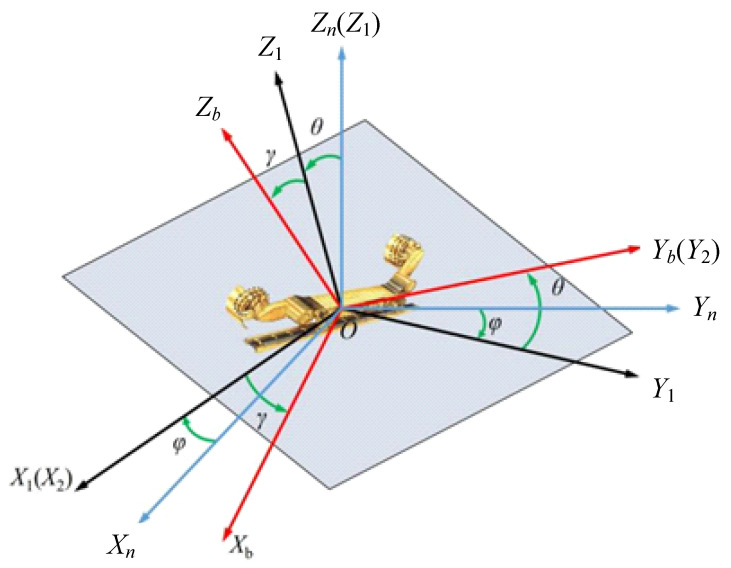
Azimuth relation between carrier coordinate system and navigation coordinate system.

The inertial components of a strapdown inertial navigation system measure the angular velocity and acceleration of the coal miner relative to an inertial frame of reference. Subsequently, the operation posture information of the mining equipment is derived through iterative calculations [9]. The perception of the mining equipment’s operational posture occurs within the body coordinate system (body frame), which cannot be directly used for calculating the vehicle’s operating attitude; calculations must be performed in the navigation coordinate system (navigation frame). The vehicle’s attitude essentially reflects the spatial relationship between the body coordinate system and the navigation coordinate system. Therefore, to determine the vehicle’s attitude, a transformation between the body and navigation coordinate systems is required. The spatial relationship between these two coordinate systems is illustrated in Figure 4, where φ, θ and γ represent the vehicle’s heading angle, roll angle, and pitch angle, respectively.

The attitude matrix $C_{b}^{n}$ shown in the following equation can be obtained by the relation of azimuth transformation between the two coordinate systems.

(1) $$C_{b}^{n}=\left(\begin{array}{ccc} \cos\gamma \cos\varphi +\sin\gamma \sin\theta \sin\varphi & \sin\varphi \cos\theta & \sin\gamma \cos\varphi -\cos\gamma \sin\varphi \sin\theta \\ -\cos\gamma \sin\varphi +\sin\gamma \sin\theta \cos\varphi & \cos\varphi \cos\theta & -\sin\gamma \sin\varphi -\cos\gamma \sin\varphi \cos\theta \\ \sin\gamma \cos\theta & \sin\theta & \cos\gamma \cos\theta \end{array}\right)$$

The angular velocity measured in real time by the gyroscope can be used to compute the attitude transformation matrix $C_{b}^{n}$, which in turn allows for solving and obtaining the attitude information of the mining equipment. The real-time acceleration recorded by the accelerometer represents the carrier’s coordinate system; thus, the total acceleration of the equipment in the carrier’s coordinate system can be converted into the navigation coordinate system using the attitude transformation matrix. By combining this with the initial velocity, attitude, and position of the carrier, navigation information can be integrated to obtain the operating speed and position of the mining equipment. The expression for this process is:

(2) $$x=x\left(0\right)+\int_{0}^{t}v_{en}^{nx}dt,\;y=y\left(0\right)+\int_{0}^{t}v_{en}^{ny}dt,\;\;\;z=z(0)+\int_{0}^{t}v_{en}^{nz}dt,$$

where *x*, *y* and *z* are the position coordinates of the carrier; *x*(0), *y*(0) and *z*(0) are the position coordinates of the initial input of the carrier; $v_{en}^{nx}$, $v_{en}^{ny}$ and $v_{en}^{nz}$ are the real-time speed of the carrier in each axis direction; *t* is the carrier running time.

## 3. Application of Strapdown Inertial Navigation System in Mining Equipment

This Research on inertial navigation and positioning for coal mining equipment began overseas in the mid-1980s. In 2001, Australia applied inertial navigation technology to continuous miners and introduced the intelligent longwall automation LASC (Longwall Automation Steering Committee) system. By employing military-grade high-precision fiber-optic gyroscopes and uniquely developed navigation algorithms, this system achieved accurate positioning of continuous miners and automatic alignment of the face conveyor [10,11,12]. In China, the Ningxia Hongliuwu coal mine implemented this system in their longwall face, resulting in significant improvements [13]. The average deviation in straightness of the working face decreased from 0.73 m to 0.42 m after implementation, greatly enhancing the standardized operational level of the working face.

**Figure 5:**
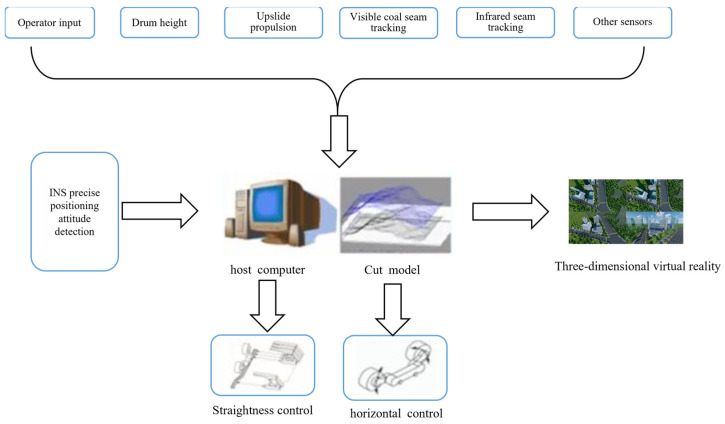
System structure of LASC.

In 2008, the LASC system underwent a technological upgrade, incorporating detailed geological data and a three-dimensional model of the mining space to enable position monitoring of production equipment in three-dimensional space, memory cutting for the continuous miner, and 3D visualization of the mined space [14]. The latest technology, LASC 2.0, includes functions such as position measurement of the continuous miner based on inertial navigation, three-dimensional space measurement of working equipment, automatic alignment of the working face, automatic height control, continuous miner identification based on infrared and visible light, and vision-based remote monitoring. This technology achieves a three-dimensional precise positioning error of ±0.1 m for continuous miners and an error of ±0.5 m for the working face alignment system, along with precise horizontal control of the working face, leading the way in achieving minimal or no human presence in coal mining operations [15]. The system structure of LASC is depicted in Figure 5.

**Figure 6:**
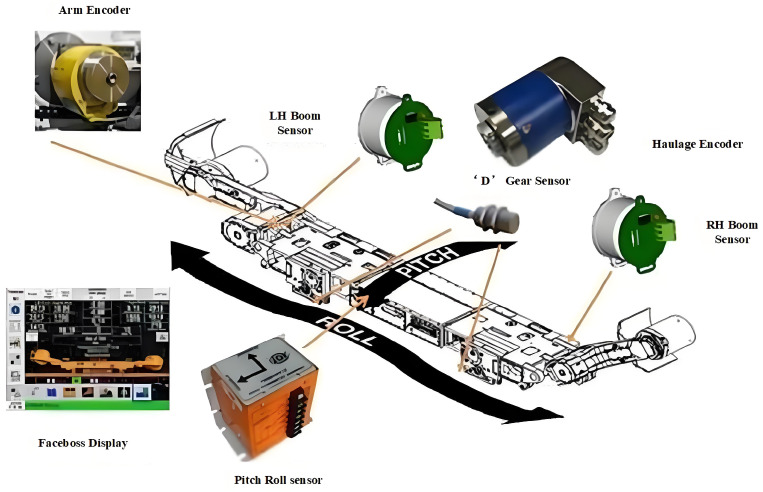
Structure diagram of Joy automatic coal cutter.

In addition to the LASC system, other foreign companies with relatively mature research include the American company JOY (Komatsu America Corp in Chicago, IL, USA) in and the German company Eickhot. JOY has developed a system called Faceboss 1.0, which is designed specifically for its intelligent coal cutters [16]. Faceboss can be directly connected to surface computer control systems, enabling remote monitoring and control of the equipment. The latest version, Faceboss 2.0, has introduced Advanced Cutter Automation (ASA), which facilitates automatic traction, horizontal control, offline graphical editing of Golp, automated extraction of triangular coal, and integrated automation of the working face. Additionally, it includes sensors such as traction encoders, tilt sensors, D-gear sensors, and body pitching/tilting sensors, with the latter serving as a backup for the INS. These sensors allow for monitoring of the cutter’s posture, position, and cutting height, enabling real-time remote control [17]. In 2013, JOY applied this technology in the extraction of medium-thin coal seams at the SNSG coal mine in Norway, offering features such as remote control of equipment, monitoring of the cutter’s pose and height, memory cutting, and path editing. They also launched the Intelligent Mining Service Center (IMSC), which achieved increased productivity and efficiency at Anglo-American operations in Australia [18]. The structure of JOY’s automated coal cutter is illustrated in Figure 6.

**Figure 7:**
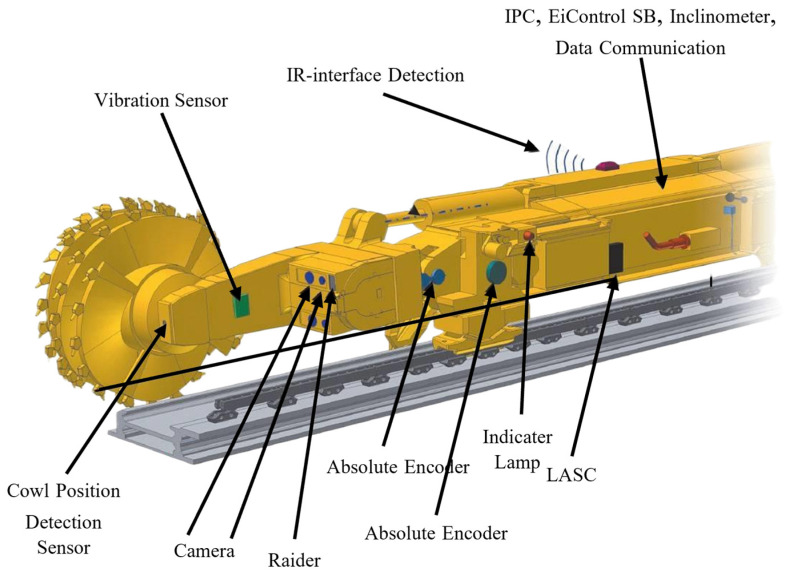
Eickhoff modern coal cutter.

Eickhoff’s coal cutter automation technology has mainly gone through four stages: Memory Cutting, EiControl, EiControlSB, and EiControlPlus. Currently, the primary system used in domestic coal cutters is EiControl, although some machines are equipped with EiControlSB. EiControlSB is an enhanced version of EiControl; officially launched in 2008 with version 1.0 and integrated with LASC functionality in 2009, the current version is 1.8. Modern Eickhoff coal cutters equipped with the EiControlPlus system are fitted with various sensors, including vibration sensors, position sensors, stroke sensors, infrared sensors, tilt sensors, and radar sensors [19,20]. Depending on different operational principles, these sensors can achieve precise monitoring of the machine’s position and orientation based on redundancy. An image of a modern coal cutter is shown in Figure 7.

The conditions for tunneling work differ significantly from those in coal mining operations. The working environment inside coal tunnels is harsher than that at the working face. Additionally, when the cutting drum is used to shear the coal wall, the machine body lacks full degrees of freedom for restraint, and the tunneling equipment is complex. Inertial sensors can only be installed on the tunneling and anchoring machine [21]. Over extended periods of operation, the drift of the inertial sensors can lead to unbounded positioning errors. Furthermore, the lack of fixed constraints between the tunneling equipment and the tunnel exacerbates the sliding of the tunneling machine, highlighting this drawback of the inertial navigation system. Therefore, when determining the pose and positioning of tunneling equipment, it is often necessary to combine other navigation methods for calibration [22].

Since the 1980s, there has been extensive research abroad on the positioning and orientation technology for cantilevered tunneling machines, with countries like Germany, the UK, and Austria leading the way in achieving results. The German company, Eickhoff, developed a system for monitoring the profile of tunneling machines and their operational status, offering four modes of operation: manual, semi-automatic, automatic, and program-controlled. The relationship between the cutter head position and the cross-section can be displayed on the workstation screen. In the UK, an instrumentation company designed an intrinsically safe computer control system specifically for tunnel boring machines. Dosco equipped a heavy tunneling machine with a cutter head positioning device that includes an inertial navigation system, enabling precise guidance of the cross-section and real-time display of cutting status. In China, institutions such as Liaoning University of Engineering and Technology, China University of Mining and Technology, and Shijiazhuang Coal Cutter Co., Ltd. have also developed automated cutting and profiling systems based on cantilevered tunneling machines for coal roadway excavation.

**Figure 8:**
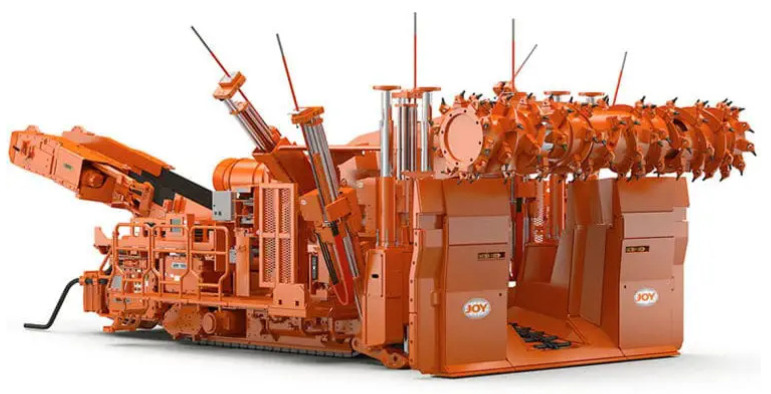
Joy’s 12ED30 anchor digger.

The University of Wollongong in Australia has conducted research on automatic anchoring and automatic mesh installation technologies, applying them to the 12ED30 model from Joy and the MB670 model from Sandvik. The specific structure of the 12ED30 boring machine is shown in Figure 8. Internationally, tunneling and full-face excavation machines feature automatic cutting technologies, equipment monitoring, and automatic control systems, enabling comprehensive remote control and cutting face monitoring. However, most of the existing automated individual machines are primarily used in non-coal mining sectors such as metal and salt mines. Currently, the technological equipment in foreign coal mine excavation operations remains semi-automated, with no complete set of intelligent rapid excavation technologies and equipment available.

**Figure 9:**
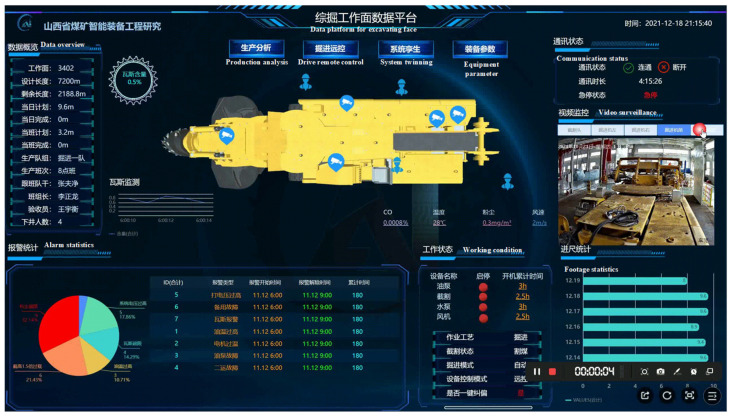
THICS Intelligent remote-control system for boring machines.

The inertial navigation system has seen widespread application in domestic tunneling equipment. In recent years, Shandong Tianhe Technology Co., Ltd. has developed the Tianhe EBZ series of tunneling and anchoring machines. This equipment is suitable for large cross-section tunnels, semi-coal rock tunnels, and rock tunnels. During drilling and anchoring operations, workers operate on a platform located beneath temporary support, effectively preventing safety incidents such as roof falls and side collapses. The company has also developed the THICSsys intelligent remote-control system for tunneling machines, which integrates various monitoring technologies, including machine vision, inertial navigation, motion control, intelligent perception, and precise positioning. Its management and control platform are shown in Figure 9. This system features comprehensive parameter sensing, state monitoring, fault diagnosis, autonomous navigation, posture correction, automatic cutting, remote control, one-click start-stop for the entire process, and multi-system integration. It enables high-precision directional control, posture adjustment, adaptive cutting, and visualization of the tunneling environment. Furthermore, it provides autonomous decision-making capabilities for the equipment cluster used in tunnel excavation, forming an intelligent tunneling operation system characterized by efficient collaboration in excavation, anchoring, support, and transport, along with automatic tunneling and intelligent control. This ultimately contributes to the creation of a digital, intelligent, and transparent working face.

## 4. Research Status of Improvement Strategy of Strapdown Inertial Navigation System in Mining Equipment

### 4.1. Limitations of Inertial Navigation Systems in Coal Mining

In the process of mining equipment operation within coal mines, precise navigation and positioning are often required. In underground mining, the navigation accuracy standards of INS involve multiple key indicators. For the main mining equipment, these include positioning accuracy, the accuracy of the heading angle of the tunneling machine, and the straightness of the working face of the coal cutter. Specifically, as shown in Table 2, the straightness refers to the deviation between the actual trajectory of the working face (such as a tunnel or mining path) and the designed trajectory. Smaller straightness errors contribute to improved mining efficiency of the mineral body [23].

When a strapdown inertial navigation system is installed on mining equipment for operation, the limited measurement accuracy of accelerometers and gyroscopes, influenced by various factors such as temperature and vibration in the complex underground environment of coal mines, can lead to sensor deviations, resulting in inaccurate trajectory calculations. Additionally, as the strapdown inertial navigation system operates over extended periods, even minor errors can accumulate over time, causing the final navigation positioning results to significantly deviate from the actual location, potentially resulting in position errors of 1 to 10 m and angular errors of 1 to 5 degrees. Due to the inability to frequently perform systematic calibration in the underground working environment, the phenomenon of drift becomes particularly pronounced, resulting in the positioning accuracy, heading angle accuracy, and linearity of the working face of mining equipment failing to meet the standards required for mining operations [24].

The drift phenomenon of strapdown inertial navigation systems leads to positioning errors, which can increase safety hazards for underground workers and consequently raise the risk of accidents. Additionally, when the positioning of mining equipment is inaccurate, it can disrupt the scheduling and transportation of resources, thereby reducing the efficiency of coal mining operations. Furthermore, as automation technology is increasingly integrated into coal mining production, the posture and positioning of mining equipment become particularly critical; the errors caused by drift phenomena will severely impact the stability of automated operations.

Due to the strong adaptability of inertial navigation in underground environments, it occupies an indispensable and important position in the positioning technology of mining equipment despite the cumulative errors caused by drift during prolonged operation. Therefore, how to compensate for the shortcomings of inertial navigation systems working underground and what improvement strategies can eliminate or compensate for these errors have become a key focus and direction of research for many experts and scholars both domestically and internationally.

### 4.2. Improvement Strategies for Strapdown Inertial Navigation Systems

The improvement strategies for strapdown inertial navigation systems mainly fall into two categories. One approach addresses the sources of navigation technology errors directly by employing zero-velocity correction techniques to rectify errors when the platform is at rest. The other approach combines inertial navigation with other navigation technologies to calibrate and correct errors. Both methods have shown promising results in enhancing performance.

#### 4.2.1. Zero Speed Correction

Among the classic error compensation methods in these improvement strategies, the zero-velocity correction technique stands out. It utilizes the speed output of an inertial navigation system when the carrier is stationary as a measure of error, enabling corrections to other types of errors. In 2019, Tian Yuan [25] applied this zero-velocity correction technique to correct errors in the inertial navigation system of a tunnel boring machine (TBM). He used the speed output from the inertial navigation system during multiple stops of the TBM as observational data for speed errors. By employing a quadratic curve for error fitting, he generated a position error curve that corrected the output position error of the inertial navigation system, thereby enhancing the positioning accuracy of the TBM. The following year, Tian conducted research on automatic positioning for the TBM using the PHINS inertial navigation system [26]. He proposed employing the zero-velocity correction method to address the issue of error drift that accumulates over time in inertial navigation. He completed industrial testing, which revealed that with a zero-velocity correction interval of 60 s, during about 2 h of testing, the TBM traveled approximately 42 m, with a cumulative northward positional deviation of about 0.249 m and an eastward positional deviation of about 0.49 m. This approach effectively met the positioning accuracy requirements for the automatic cutting operation of a TBM in a short period.

**Figure 10:**
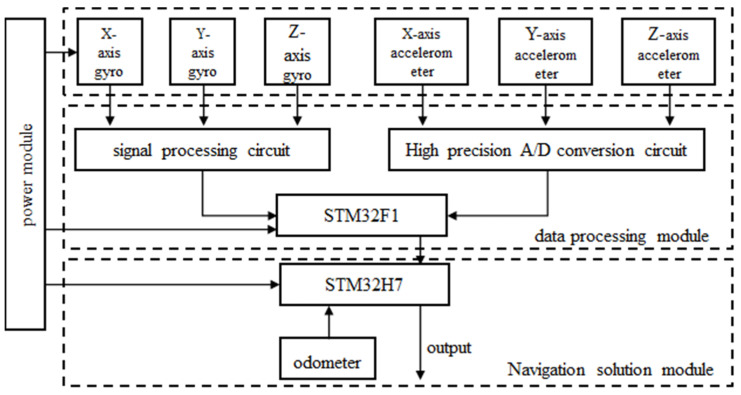
Design block diagram of the inertial navigation system of the headboring machine.

Hao Jinjie [27] and colleagues researched the issues of accumulated errors and low positioning accuracy in the navigation systems of comprehensive tunneling machines. They optimized the design of the navigation system, and the basic principle is shown in Figure 10. Based on an in-depth analysis of the inertial navigation principles of comprehensive tunneling machines, they focused on the hardware and software design of the navigation system centered around the STM32H7 controller, as well as strategies for optimizing rocking alignment and bias estimation. Finally, they completed experimental validation and analysis. The experimental results indicate that the optimized navigation system for comprehensive tunneling machines can keep the alignment azimuth angle error within 0.15° and the bias estimation error within 15%, meeting the navigation accuracy requirements for tunneling machines.

Sun Wei et al. [28] developed a closed-loop Kalman filtering method with zero-velocity correction based on the unique motion working mode of end-loading coal cutters to calculate inertial navigation attitude and correct the bias information of inertial devices. The zero-velocity correction technique requires the carrier to stop periodically to correct various errors, which greatly limits its application. In response, Fang Jing et al. [29] proposed a dynamic zero-velocity correction technique that uses the condition of zero velocity in the lateral and vertical directions of the vehicle coordinate system as a constraint. They constructed a virtual noise observation and designed a Kalman filtering method with U-D covariance decomposition and sequential processing for real-time trajectory correction.

Wang Shijia [30] and others established a Kalman filtering model based on the non-complete constraint conditions of the inertial navigation attitude error equation and dynamic zero-velocity correction technology. They used a mobile platform to simulate the operational trajectory of the coal cutter, resulting in a 30% improvement in positioning accuracy.

**Figure 11:**
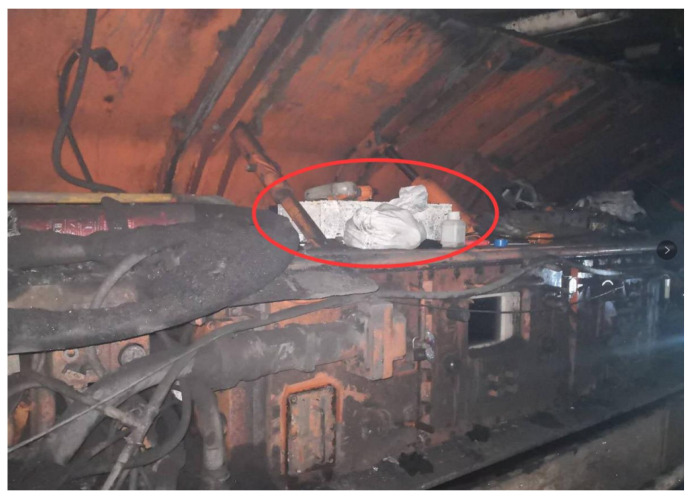
Cutter strapdown inertial navigation system site layout.

Wu Gang [9] and others have proposed using an optimized Unscented Kalman Filter (UKF) algorithm for initial alignment in strapdown inertial navigation systems based on the actual motion states of a coal cutter to improve alignment precision. They developed and simulated a dynamic model for the vibration of the coal cutter, effectively using the rotation vector method to mitigate the impact of vibrations on the accuracy of the inertial navigation system. In practical mining operations, Wu Gang and his team applied their optimized UKF algorithm to the JOY LWS830-07LS06C long-arm coal cutter in a particular mining face, as illustrated in Figure 11. Through a single-axis rotation modulation scheme with optimal parameters, they achieved a reduction in positioning error of approximately 93% compared to the error compensation prior, significantly enhancing the precision of the coal cutter’s operational attitude perception.

**Figure 12:**
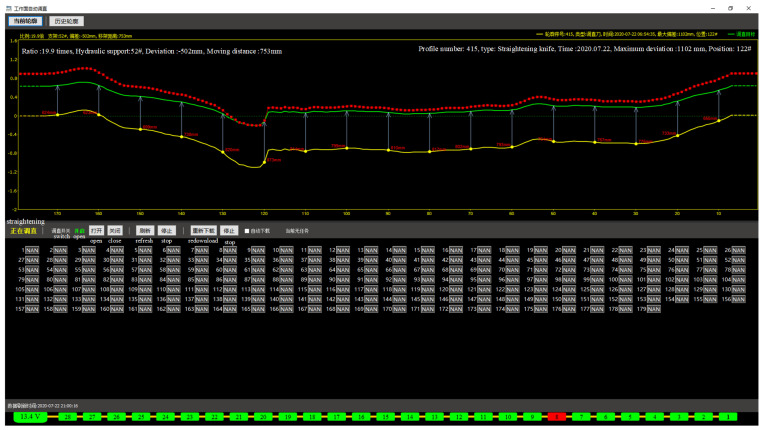
Comparison of straightening of scraper conveyor before and after error compensation.

As shown in Figure 12, before error compensation, the scraper conveyor deviated from the reference line by a maximum of 110 cm and was offset from the coal cutter’s cutting depth by 30 cm. After an error compensation, the average deviation of the scraper conveyor from the reference line was reduced to 80 cm, with an average offset from the coal cutter’s cutting depth of 2 cm. The straightening error of the scraper conveyor after compensation, which reflects the planar positioning error, is about 17% of the pre-compensation value, indicating a significant improvement in the positioning accuracy of the coal cutter’s inertial navigation system.

#### 4.2.2. Integrated Navigation

Due to issues with inertial navigation that cannot be effectively resolved in the navigation of mining equipment, many scholars have proposed combining inertial navigation with other navigation methods. This includes integrating inertial navigation with wireless sensor networks, GPS, total stations, odometers, machine vision, and laser radar in order to achieve more precise navigation and positioning for mining equipment.

**Figure 13:**
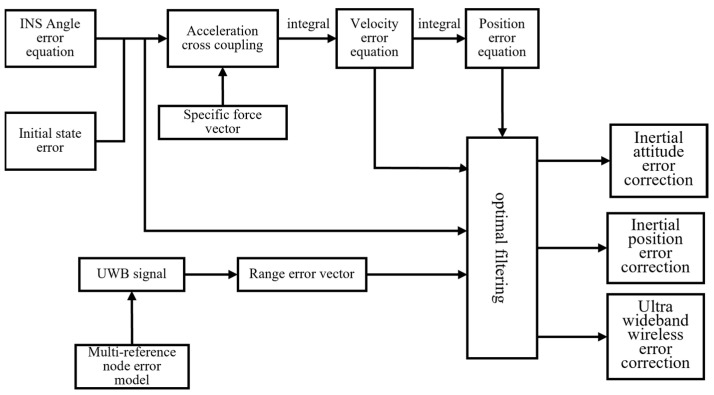
Basic principle of INS/UWB dual system cooperative positioning model.

Based on the established inertial coordinate system navigation model for mining machines created by Fan Qigao [31], Li Wei [32], and others, a simulation analysis of inertial navigation positioning was conducted. By introducing ultra-wideband (UWB) wireless sensor network positioning technology, a collaborative positioning model for the INS/UWB dual system was developed, as shown in Figure 13. This model results in a dynamic tracking error for the machine’s orientation that is less than 0.7° and a positional dynamic tracking error of less than 20 cm, meeting the positioning requirements for mining machines in closed coal mine environments. The error angular rates from the inertial navigation system are integrated once and combined with the initial biases, along with the spatial state errors of the mining machine, to derive the error angles $∆\phi_{E}$, $∆\phi_{N}$ and $∆\phi_{U}$. This leads to cross-coupling errors in the acceleration measurements, which, when adjusted for the accelerometer’s zero bias error, produce acceleration errors $∆a_{E}$ and $∆a_{N}$. Further integration yields velocity errors $∆V_{E}$ and $∆V_{N}$ and navigation position errors ∆λ and ∆L. On the UWB side, by combining the error model of multiple reference nodes, the distance error vector $∆P_{UWB}$ between reference and mobile nodes is obtained. After comprehensively analyzing and considering the relationship of system error transmission, equations for errors at each stage are established, and an optimal filtering strategy is employed to correct the inertial attitude, position, and UWB ranging errors within the collaborative positioning system, ensuring that the attitude dynamic tracking error remains below 0.7° and the positional dynamic tracking error stays below 20 cm, thereby satisfying positioning requirements in closed coal mine environments.

**Figure 14:**
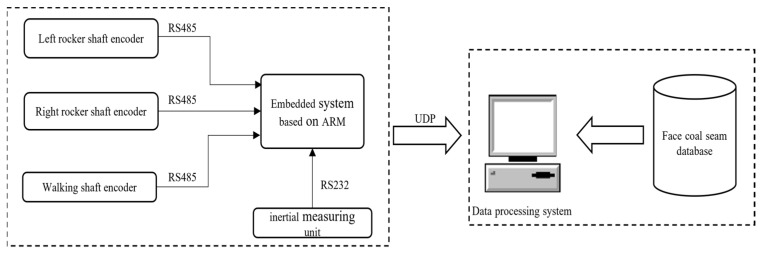
Principle of position and posture monitoring device of a coal miner.

Ge Shirong, Wang Shibo, and Wang Shijia, among others [33], have developed a coal cutter pose monitoring device based on a GIS of the working face, which is shown in Figure 14, using a method that integrates inertial navigation with a geographic information system database. This device can monitor the operational trajectory of the coal cutter in real time and its relationship with the coal seam roof and floor. It achieves three-dimensional positioning of the mining machine in the working face and monitors geological information of the coal seam, providing strong support for the automatic adjustment of the mining machine’s height based on geological conditions.

**Figure 15:**
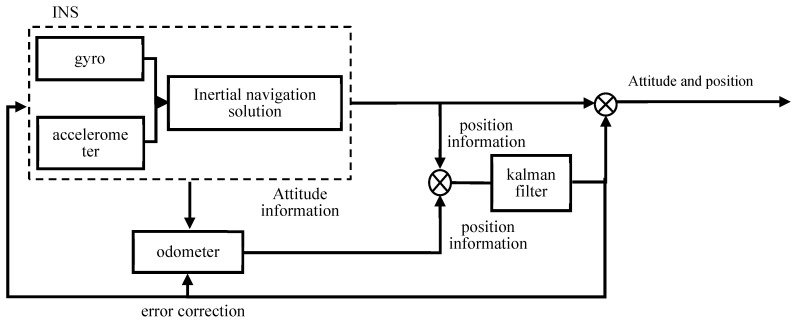
“Inertial navigation + odometer” working principle.

In 2021, Shi Jinlong et al. [34] proposed a positioning method for coal cutters that combines inertial navigation and odometry. This method involves installing a strapdown inertial navigation system inside the coal cutter and connecting the odometer to the pinion axle of the machine. By calculating the pose information of the coal cutter using inertial navigation and fusing positional data from the inertial system and the odometer through Kalman filtering, the method corrects the error parameters of both systems using closed-loop feedback, ultimately outputting the pose of the mining machine. The specific principles are illustrated in Figure 15. This positioning approach ensures that the locating error of the coal cutter in complex working environments is less than 5 cm, meeting the precision requirements for mining operations.

HAN et al. [35] also proposed a fully damped inertial navigation/odometry combination method based on underground excavation equipment to provide long-term high-precision positioning and navigation information. This system maintains high-precision directional measurements for a week or longer, even in the presence of sensor drift errors and odometer inaccuracies. Field tests have shown that this method can achieve a maximum heading error of 0.057°, a maximum pitch error of 0.004°, and a roll error, effectively meeting the high-precision navigation requirements for long-term underground excavation in severely sliding and vibrating environments.

**Figure 16:**
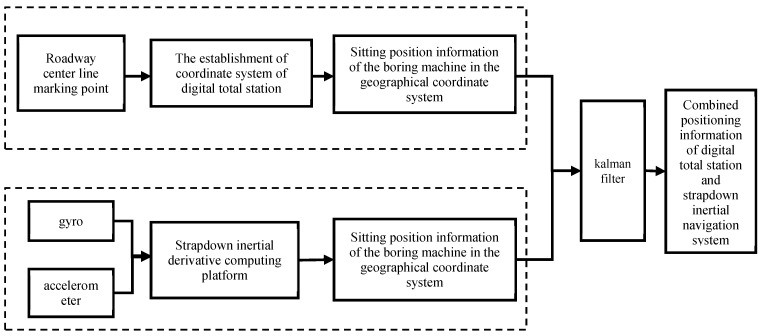
Based on the combined positioning and orientation principle of optical fiber inertial navigation and digital total station.

In 2022, Ma Hongwei and others [36] analyzed the combined positioning and orientation principles of fiber optic inertial navigation and digital total stations. They established error models for both the fiber optic inertial navigation system and the digital total station, along with the state equations and measurement equations for the integrated positioning and orientation system. By employing a Kalman filter, they predicted and estimated the state variables and measurements of the combined system while considering the impact of digital total station positioning accuracy on the fusion results. This led to the acquisition of integrated information for the autonomous positioning and orientation of tunneling machines, as illustrated in Figure 16.

They built a testing platform for the positioning and orientation of tunneling machines and conducted positioning and orientation tests using a self-localization method combining inertial navigation and total stations, simulating the operation of a tunneling machine along the centerline of a tunnel. The test results showed that when determining the measurement noise variance (R) based on the positioning accuracy of the digital total station, the combined positioning and orientation precision were maximized. The maximum positioning errors were 3 cm in the *x*-axis direction and 2 cm in the *y*-axis direction, while the orientation errors for heading, pitch, and roll angles were all less than 0.005°, meeting the technical requirements for the positioning and orientation of tunneling machines in coal mines.

Huang Dong [37] et al. and Yang [38] et al. both proposed combining visual measurement and inertial navigation, using a laser strapdown inertial navigation system to obtain the attitude information of the tunneling machine, while visual technology is used to acquire the position information of the tunneling machine, achieving real-time measurement of the pose parameters of the tunneling machine. However, due to the non-horizontal road surface in this study, the motion analysis of the established rigid body model of the tunneling machine is not precise enough; the accuracy of position measurement using monocular vision is relatively low, and further analysis of the sources of measurement errors is needed.

In 2018, Mao Qinghua [39] et al. designed a sensing detection system to detect the pose of both the cutting head and the body of the tunneling machine. They utilized cylinder stroke sensors to detect the pose of the cutting arm, achieving pose detection of the cutting head. For body pose detection, they combined laser and ultrasonic sensors with inertial navigation and used a geomagnetic sensor for calibration. In this combined method, the inertial navigation signals are not shielded by the explosion-proof casing, addressing the issue of cumulative errors in heading angle due to changes over time, which reduced the heading angle error of the tunneling machine to within 0.2 degrees.

In 2019, Wang Yizhong [40] et al. assembled a set of industrial cameras and a strapdown inertial navigation system in a coaxial reverse configuration on the body of the tunneling machine to create a deviation measurement unit. This unit measures the spatial vectors of the laser targeting instrument’s light source spot, the spatial vectors of the tunnel cross-section spot, and the pose information of the tunneling machine. By combining visual measurement with inertial navigation technology, they established spatial vector constraint equations and coordinate transformations to obtain the position information of the tunneling machine, realizing real-time pose measurement of the tunneling machine. This pose measurement method can provide six degrees of freedom information for the real-time pose of the tunneling machine, which can be used for posing measurement in coal mine tunneling machines. However, due to the short length of the deviation measurement unit, errors may occur in the calculated spatial vectors, affecting the accuracy of position measurements.

**Figure 17:**
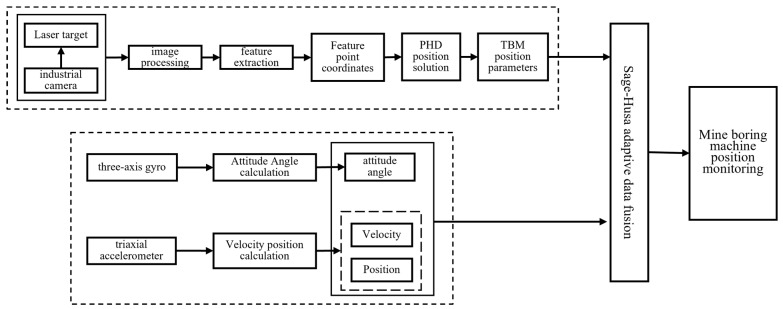
Inertial navigation and “vision + laser target” working principle.

In 2020, Zhang Chao [41,42] et al. proposed a combination of binocular vision and strapdown inertial navigation for positioning, using a rotation vector algorithm to solve for the inertial pose. They constructed a measurement target using infrared LED light sources, extracted features from the center points of the light spots and achieved continuous measurement through the alternating movement of dual targets. A 3D-3D motion estimation method was employed to complete the pose parameter solution. The least squares method was used for data alignment, and Kalman filtering was implemented for data fusion, enabling accurate measurement of the tunneling machine’s pose, as illustrated in Figure 17. This positioning method resulted in a measurement error of 0.03 m in the X direction and a heading angle error of less than 1°. In 2024, Mao Qinghua [43] et al. similarly adopted a positioning scheme that combined inertial navigation with “vision + laser targets”. Building on this, they proposed a fusion method based on Sage–Husa adaptive filtering for inertial and visual information, resulting in maximum errors of 0.029°, 0.051°, and 0.0113° for pitch, roll, and heading angles, respectively. The position error in the tunnel width direction within a range of 30 m was within 0.033 m, and the position error in the tunneling direction was within 0.062 m, meeting the precise positioning requirements for underground mining equipment.

In the current status of improved applications of inertial navigation in mining equipment, both zero-velocity correction technology and integrated navigation technology require algorithms capable of multi-information fusion to achieve accurate positioning of the carrier. The Kalman filtering algorithm is used in zero-velocity correction, while neural network algorithms, fuzzy adaptive algorithms, and others are employed in integrated navigation. These algorithms are central to establishing inertial navigation error compensation models. However, there is relatively less research on improved fusion algorithms for inertial navigation in mining equipment compared to other industrial fields. This paper suggests that future research should focus on these fusion algorithms.

## 5. Conclusions

(1) The strapdown inertial navigation system, as a relatively advanced navigation and positioning system, possesses several advantages, including strong anti-jamming capability, compact size, lightweight, high perception accuracy, high reliability, good real-time performance, and independence from external information. It can achieve real-time automated remote attitude positioning and has been successfully applied to the attitude positioning of mining equipment in comprehensive tunneling and mining faces. This paper systematically introduces the current widely used inertial navigation positioning technology in mining equipment from technical principles, practical applications, and improvement strategies.

(2) Mining and excavation projects often feature characteristics such as wide coverage, strong concealment, harsh environments, high dynamic response requirements, and long positioning cycles. In actual production activities, the appropriate attitude positioning technology is usually selected based on the positioning goals and the environment. This paper analyzes commonly used attitude positioning technologies in mining engineering, including GPS positioning, visual positioning, and laser positioning. It elaborates on the unique advantages of strapdown inertial navigation systems in the application of mining equipment.

(3) This section outlines the development history of strapdown inertial navigation systems in mining equipment and analyzes their important roles in memory cutting, working face straightening, and precise positioning of excavation equipment. Moreover, due to the complex environment of the tunneling working face and the extended working time, the inherent shortcomings of strapdown inertial navigation systems, particularly the cumulative positioning errors that arise from a prolonged operation, are also revealed. Therefore, the application of strapdown inertial navigation systems in mining equipment requires significant improvements and refinements.

(4) This section analyzes the improvement strategies for strapdown inertial navigation systems in addressing cumulative errors. Firstly, error correction during the carrier’s zero-velocity state is achieved through secondary fitting calculations and auxiliary computations using STM32 microcontrollers to implement corrections in the zero-velocity state. Additionally, the introduction of dynamic zero-correction technology allows for error observation and correction without requiring the carrier to be in a stationary state. This is accomplished through algorithms such as Kalman filtering to correct the errors inherent in the inertial navigation system itself.

(5) In terms of combined navigation technology that integrates strapdown inertial navigation systems with other attitude positioning technologies, this section explores the combination of strapdown inertial navigation systems with wireless sensor networks, GPS systems, total stations, and visual positioning technologies. This approach aims to leverage the strengths of each technology to compensate for its weaknesses, thereby achieving higher precision and greater efficiency in attitude positioning.

Currently, the research and application of strapdown inertial navigation systems in mining equipment are still in the early research stage and are far less developed and precise compared to applications in other industrial fields. This is largely due to the complexity of the mining work environment. In the improvement strategies, whether it is zero-velocity correction technology or combined navigation technology, the calculation and integration of algorithms are essential. Therefore, to achieve a more precise attitude positioning of excavation equipment, it is necessary to conduct in-depth research on algorithms that can be applied to zero-velocity correction and combined navigation technology, which are both more accurate and practical for real-world applications. The algorithms and integrated navigation research presented in this paper can provide ideas and method support for future work. This ongoing effort will continuously enhance the precision of strapdown inertial navigation systems and improve the effectiveness of attitude positioning in mining equipment.

## Figures and Tables

**Table 1:** Comparison of positioning technology of mining equipment.

Name	Advantage	Deficiency	Application Scenarios
GPS positioning system	High precision, real-time and global coverage	Easy to be affected by the environment, limited accuracy and high power consumption	Laboratory test and field test
Inertial navigation and positioning	Strong independence and anti-interference, high precision and real-time	The error accumulates, and the algorithm is complicated in the long-time navigation	Laboratory test and field test
laser positioning	High precision and fast measurement speed	Easily disturbed by external factors and requires regular maintenance	Laboratory test and field test
visual positioning	High flexibility, low cost, high precision	Easily affected by the environment, and the algorithm is complex	Laboratory test and field test
RFID orientation	No visual distance is required, and multi-target positioning can be achieved	The precision is limited, and the environmental adaptability is poor	Field test

**Table 2:** Standards for Positioning and Orientation of Mining Equipment.

Expected Error	Positioning (cm)	Course Angle (°)	Straightness of the Working Face (°)
Heading machine	±10	±1	
coal cutter	±5~15	±1	±1

## Data Availability

All data and code used or analyzed in this study are available from the corresponding author on reasonable request.
